# Evaluation of improved coloured targets to control riverine tsetse in East Africa: A Bayesian approach

**DOI:** 10.1371/journal.pntd.0009463

**Published:** 2021-06-21

**Authors:** Roger D. Santer, Michael N. Okal, Johan Esterhuizen, Steve J. Torr

**Affiliations:** 1 Institute of Biological, Environmental, and Rural Sciences, Aberystwyth University, Aberystwyth, United Kingdom; 2 International Centre of Insect Physiology and Ecology, Nairobi, Kenya; 3 Liverpool School of Tropical Medicine, Liverpool, United Kingdom; CIRAD, FRANCE

## Abstract

**Background:**

Riverine tsetse (*Glossina* spp.) transmit *Trypanosoma brucei gambiense* which causes Gambian Human African Trypanosomiasis. Tiny Targets were developed for cost-effective riverine tsetse control, and comprise panels of insecticide-treated blue polyester fabric and black net that attract and kill tsetse. Versus typical blue polyesters, two putatively more attractive fabrics have been developed: Vestergaard ZeroFly blue, and violet. Violet was most attractive to savannah tsetse using large targets, but neither fabric has been tested for riverine tsetse using Tiny Targets.

**Methods:**

We measured numbers of *G*. *f*. *fuscipes* attracted to electrified Tiny Targets in Kenya and Uganda. We compared violets, Vestergaard blues, and a typical blue polyester, using three replicated Latin squares experiments. We then employed Bayesian statistical analyses to generate expected catches for future target deployments incorporating uncertainty in model parameters, and prior knowledge from previous experiments.

**Results:**

Expected catches for average future replicates of violet and Vestergaard blue targets were highly likely to exceed those for typical blue. Accounting for catch variability between replicates, it remained moderately probable (70–86% and 59–84%, respectively) that a given replicate of these targets would have a higher expected catch than typical blue on the same day at the same site. Meanwhile, expected catches for average violet replicates were, in general, moderately likely to exceed those for Vestergaard blue. However, the difference in medians was small, and accounting for catch variability, the probability that the expected catch for a violet replicate would exceed a Vestergaard blue equivalent was marginal (46–71%).

**Conclusion:**

Violet and Vestergaard ZeroFly blue are expected to outperform typical blue polyester in the Tiny Target configuration. Violet is unlikely to greatly outperform Vestergaard blue deployed in this way, but because violet is highly attractive to both riverine and savannah tsetse using different target designs, it may provide the more suitable general-purpose fabric.

## Author summary

Riverine tsetse flies transmit parasites that cause sleeping sickness, so Tiny Targets have been developed to control them. Tiny Targets comprise adjacent panels of insecticide-coated blue polyester fabric and black net, and they work because tsetse are attracted by the blue fabric, and are killed by the insecticide if they make contact. In order to improve Tiny Targets, new coloured polyester fabrics have been developed with the aim of attracting more tsetse. These include a blue fabric produced by Vestergaard S.A. and currently used to make Tiny Targets, and a violet fabric developed by modelling fly colour vision and effective against savannah tsetse. We recorded the numbers of tsetse attracted to Tiny Targets made from these fabrics, and a typical blue polyester. Based on our data, we predicted the performance of such Tiny Targets in future deployments. It is highly probable that both Vestergaard blue and violet fabrics will attract more tsetse than typical blue. The average catch at a violet Tiny Target is quite likely to exceed that at a Vestergaard blue one, but because the difference is small and catches variable, there is unlikely to be a meaningful performance difference between these fabrics when deployed this way.

## Introduction

Riverine tsetse are the vectors of *Trypanosoma brucei gambiense* which causes Gambian Human African Trypanosomiasis (gHAT, or Gambian sleeping sickness). Vector control can make a vital contribution to current efforts to eliminate this disease, provided that control devices are cost effective [1]. To this end, Tiny Targets have been developed that consist of two adjacent 25 cm x 25 cm panels of insecticide-impregnated blue polyester fabric and black polyester net, respectively [2–4]. Tsetse are attracted to the coloured panel and are killed upon contacting the target. Polyester fabrics are used to construct Tiny Targets because they are more durable than the phthalogen blue cottons that were used in traditional trap and target designs. Against this practical advantage, the blue polyesters tested so far have not been as attractive to tsetse as phthalogen blue cottons [4]. Thus, there has been an opportunity to further improve Tiny Targets by modifying the colour of the polyester fabric panel.

Currently, two families of putatively improved polyester fabrics exist. Based on the long-established principle that blue targets are highly attractive to tsetse, and that suppressing reflectivity at UV wavelengths enhances a target’s attractiveness [4–7], blue fabrics developed by Vestergaard S.A. are currently used in the ZeroFly Tiny Targets produced by that company. Alongside these, violet polyesters have been developed by taking a subtly different approach, wherein fly photoreceptor signals were calculated for previously tested fabrics and statistically related to the numbers of tsetse they caught, leading to the principles that signals in a UV-Blue sensitive photoreceptor (R7y) tend to enhance attraction, and signals in a UV (R7p) and a green sensitive photoreceptor (R8y) tend to reduce attraction [8,9]. The violet fabric was developed by identifying a dye that would more effectively exploit these principles [10]. Both fabrics were evaluated against savannah tsetse in Zimbabwe using the large target size recommended for these flies, wherein the violet fabric often achieved significantly greater catches than a black cotton standard target, whilst the Vestergaard blue fabric did not [10]. Neither fabric has yet been evaluated for use against riverine tsetse using the Tiny Target configuration.

Experimental tests to evaluate the relative effectiveness of putatively improved Tiny Target designs for riverine tsetse are problematic. Small improvements in the cost-effectiveness of Tiny Targets might be meaningful when they are deployed at scale, but gaining experimental proof of such effects using the kinds of statistical tests traditionally used by biologists requires a very large sample size. Thus, it may be costly, difficult, or even impossible to accrue an adequate sample size in a single experiment to provide robust statistical proof for an improved Tiny Target design. Bayesian statistical methods are an approach that can help overcome some of these difficulties. Bayesian methods have the potential to incorporate prior knowledge from earlier experiments to allow for an accumulated understanding across multiple studies, rather than requiring definitive experimental proof within any single one [11]. Bayesian statistics estimate the most plausible probability distributions for model parameters (e.g. treatment effects) that are consistent with prior expectations and experimental data, meaning that they explicitly represent and account for the degree of uncertainty in these parameters [11]. Finally, rather than providing a p value that can be used to evaluate a null hypothesis, Bayesian methods facilitate the simulation of future events, given the entire plausible distributions of model parameters, so the probability of a situation of interest occurring (e.g. one target outperforming another), can be directly computed from these simulations, providing an intuitively understandable evaluation of treatment effects that accounts for uncertainty in statistical model parameters [11].

In this work we evaluate catches of the riverine tsetse *Glossina fuscipes fuscipes* from Tiny Targets in Kenya and Uganda, testing putatively improved Vestergaard blue and violet fabrics, as well as a typical blue polyester similar to those evaluated when Tiny Targets were originally developed [4]. We use a Bayesian approach to model tsetse catches recorded at the different Tiny Target designs, generating posterior distributions for model coefficients that capture uncertainty in them. Using these models, we simulate 10,000 future replicates of each Tiny Target design that capture uncertainty in treatment effects and variability across sampling days and locations. We then employ probabilistic reasoning to evaluate whether a given design is likely to outperform another in future deployments.

## Methods

### Fabrics

**Fig 1:**
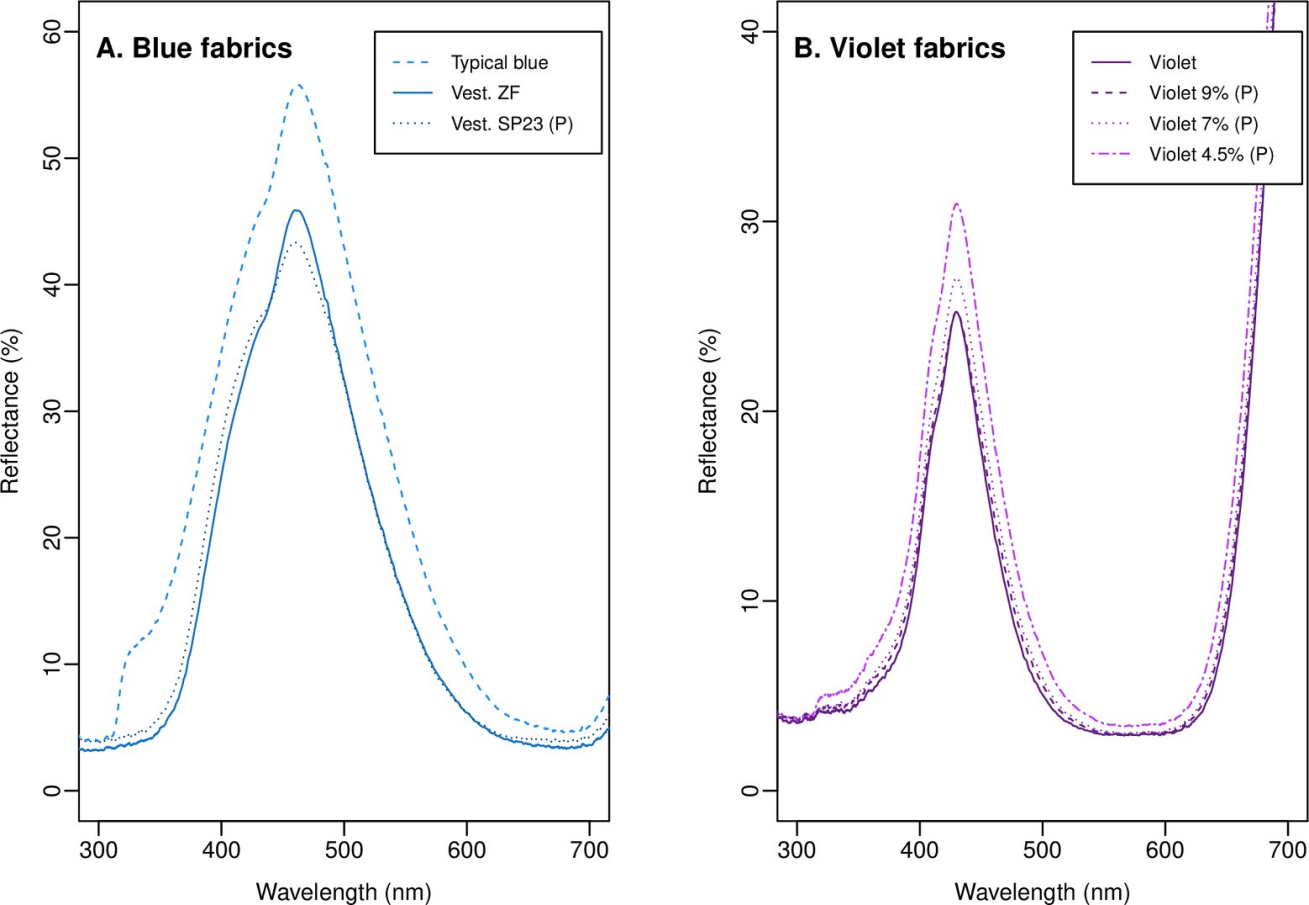
Reflectance spectra for the polyester fabrics tested in this work. (A) Typical blue is representative of the blue polyesters tested by [4]. Vest. ZF is the Vestergaard S.A. fabric currently used in the production of ZeroFly Tiny Targets, and Vest. SP23 (P) is a prototype variation on that fabric. (B) Violet is the improved violet fabric tested against savannah tsetse in Zimbabwe by [10]. The remaining violets designated (P) are unset prototypes produced using varying depths of the same dye. Spectra were measured using an Ocean Optics USB4000 spectrometer, PX-2 pulsed xenon light source flashing with a 30ms period, reflectance probe, and WS-1-SL standard (Ocean Insight Inc., Orlando, FL, USA). The reflectance probe was angled 45° to vertical and its tip was positioned 6 mm from the sample. A 120 ms integration period, boxcar width of 5, and 25 scans to average were used. Three replicate spectra were recorded for each of two reflectance probe azimuth angles on one side of the fabric. These replicates were averaged to produce the plotted functions.

The reflectance spectra for the fabrics investigated in this study are shown in Fig 1. We tested three blue fabrics (Fig 1A), comprising: a typical blue polyester similar to those originally tested by [4] and identical to that tested by [10] (typical blue; produced using CI Disperse Blue 60 at a dye bath concentration of 2.5%); the blue fabric currently produced by Vestergaard S.A. for its ZeroFly Tiny targets (Vest. ZF), also tested in [10]; and a prototype blue fabric that was produced by Vestergaard S.A. (Vest. SP23 (P)). We also tested a number of violet fabrics (Fig 1B): our original violet fabric as tested against savannah tsetse in Zimbabwe [10] (violet; produced using CI Disperse Violet 57 at a dye bath concentration of 7%); and a number of violet fabrics with varying depths of the same dye that were produced as small samples and were unset, resulting in more crinkled fabrics (labelled (P) for prototype to separate them from the fabric previously tested). Based on our prior analysis of fly photoreceptor signals [8,9], we expected tsetse attraction to increase with dye concentration among these three prototype violet fabrics. All violet and typical blue fabrics used the same base polyester (Jupiter polyester microfibre with density 78 decitex and 72 filaments, and a weight of 80g/m^2^; Toray Textiles Europe Ltd., Mansfield, UK). Both Vestergaard S.A. blue fabrics used the same base polyester which had a coarse surface texture in contrast to the smooth surface of the other fabrics.

### Tsetse catch data

We conducted a total of three experiments at study locations selected in order to sample the riverine tsetse *Glossina fuscipes fuscipes*, which is among the most important vectors of Gambian sleeping sickness.

We conducted two experiments on Chamaunga Island, Lake Victoria, Kenya, which was the site of the original work evaluating coloured Tiny Targets against riverine tsetse [4]. The first of these evaluated violet, Vest. ZF, Vest. SP23(P), and typical blue fabrics, and the second evaluated Vest. ZF and the three prototype violets. Flies were sampled using grids of electrocuting wires overlaying the Tiny Target that operated between 09.00 and 12.00 hours on each sampling day. Each experiment consisted of four repeated Latin squares (four fabrics x four sites x four days), comprising 64 target deployments in total (16 deployments per target).

We conducted a third experiment in the Arua district of northern Uganda, testing Vest. ZF, violet, and typical blue fabrics. In this experiment, flies were sampled between 09.00 and 13.00 hours on each sampling day. The experiment consisted of six repeated Latin squares (three fabrics x three sites x three days), though the number of flies caught was low and, as a result, data collection was terminated after a total of 50 target deployments (16–17 deployments per target) with the final Latin square incomplete.

For each experiment, we analysed total catches of *G*. *f*. *fuscipes* on each sampling day, comprising both sexes, and flies recovered from both the fabric and net portions of a Tiny Target.

### Statistical analysis

We applied Bayesian statistical methods using the Rethinking package for R and Stan [11]. We used R version 3.6.3 and Stan version 2.19.1. The inclusion of prior information in Bayesian statistical models is a feature of them that contrasts with the statistical methods more commonly applied by biologists. Therefore, we divide our analyses into two sections: (i) an analysis of Tiny Target performance that did not employ informative priors, and (ii) a fly photoreceptor signal-based analysis that used informative priors. For the benefit of readers more familiar with a frequentist statistical approach, we provide such tests in S1 Statistics.

#### An analysis of Tiny Target performance without informative priors

Here we provide a detailed explanation of the statistical models used to evaluate our Kenya experiments and will subsequently explain how this approach was modified for other contexts. These models had the following general form:

$$\begin{array}{l} total\;catch∼Poisson(\lambda )\\ log(\lambda )=\alpha +{z\alpha }_{rep[i]}\times \sigma_{rep}+{z\alpha }_{day[i]}\times \sigma_{day}+{z\alpha }_{site[i]}\times \sigma_{site}+\beta_{target1}\times Target1+\beta_{target2}\times Target2+\beta_{target3}\times Target3\\ \alpha ∼Normal(0,10)\\ \beta_{target1}∼Normal(0,10)\\ \beta_{target2}∼Normal(0,10)\\ \beta_{target3}∼Normal(0,10)\\ {z\alpha }_{rep}∼Normal(0,1)\\ {z\alpha }_{day}∼Normal(0,1)\\ {z\alpha }_{site}∼Normal(0,1)\\ \sigma_{rep}∼HalfNormal(0,1)\\ \sigma_{day}∼HalfNormal(0,1)\\ \sigma_{site}∼HalfNormal(0,1) \end{array}$$

We modelled tsetse catches at Tiny Targets as a Poisson process (line 1), in which the expected catch, λ, was the log of a linear model containing a variety of random and fixed factors (line 2). Because tsetse catches were over-dispersed relative to the Poisson expectation, our linear models included a random intercept for each replicate in our dataset (i.e. each individual recorded daily catch; zα_rep[i]_ × σ_rep,_, line 2) to explicitly model the overdispersion of tsetse catches (see also S1 Statistics). In this analysis, a dummy variable was assigned to each target under test except for Vest. ZF (Target1-3, line 2), and the linear model included a coefficient for each that estimated differences in catches from that standard target (β_target1-3_, line 2). The linear model had a hierarchical structure to model random intercepts for each sampling day and site in order to account for clustering in the data, and a random intercept for each catch replicate as previously described (line 2). This was done using a non-centred parameterisation wherein the model estimated the variance within a given cluster type (σ_day_, σ_site_, σ_rep_), and a z score for each member of a cluster (zα_day[i]_, zα_site[i]_, zα_rep[i]_). Such a parameterisation was necessary for efficient model fitting (see also [11]). Bayesian analyses can incorporate ‘priors’ based upon previous knowledge and expectations, but in this analysis we used regularising priors only. These are priors that helped the models to be efficiently fitted by indicating that extreme values were unlikely, but provided no strong or directional expectations that would impact the outcomes of the analysis. The distributions, means, and standard deviations of the priors used for each parameter in this model are specified on lines 3 to 12. Model fitting itself was carried out using the map2stan() function of the Rethinking package, using three Markov chains with 10,000 iterations each. We report efficiency and convergence statistics for these chains when we report these models: very low effective sample sizes (N_*effective*_) and $\hat{R}$ >1.0 would indicate fitting problems, but none were apparent in the reported analyses. Posterior predictive plots for model validation are provided in S1 Statistics.

We caught few tsetse during our experiments in Uganda, and recorded zero catches in 38% of target replicates. Since zero catches might have resulted from there being no flies in the vicinity of a target (structural zeroes), or from no flies being attracted to a target (sampling zeroes), we applied a zero-inflated model in addition to a model like that described above. The zero-inflated model had the following adjustment to the above form (the remaining priors were identical and have not been duplicated here):

$$\begin{array}{l} total\;catch∼ZIPoisson(p,\lambda )\\ logit(p)=\alpha_{p}\\ log(\lambda )=\alpha +{z\alpha }_{rep[i]}\times \sigma_{rep}+{z\alpha }_{day[i]}\times \sigma_{day}+{z\alpha }_{site[i]}\times \sigma_{site}+\beta_{target1}\times Target1+\beta_{target2}\times Target2\\ \alpha_{p}∼Normal(0,1) \end{array}$$

In this model, the probability of a zero catch being recorded is first estimated using a binary logistic process representing the probability that there were no tsetse in the vicinity of a given target on a given sampling day. Subsequent to this, an over-dispersed Poisson model is fitted that is identical to that described above with the exception that only two Target dummy variables were included in this analysis since only three targets were tested in each Latin square (line 3).

#### A fly photoreceptor signal-based analysis using informative priors

The putatively improved fabrics tested in this work were designed rationally with that intention, and thus prior knowledge clearly formed the basis for our work but was not represented in the above analysis. The design of our violet fabric was based upon fly photoreceptor signals, which can be calculated from fabric reflectance spectra, and provide a discrete set of colour metrics that describe fabric colour properties relevant to flies [8–10]. We thus extended our analyses to incorporate these aspects.

Prior to our work, a large dataset of *G*. *f*. *fuscipes* catches at 37 differently coloured Tiny Targets had been published, comprising 15 separate experiments conducted at the same Kenyan study location as our experiments [4]. By calculating the fly photoreceptor signals that would be elicited by the fabrics used in that study, we previously estimated the ways in which those photoreceptor signals related to the tsetse catches reported, and these models were used to guide the design of our violet fabric [8–10]. Thus, we had a clear prior expectation of how fly photoreceptor signals would affect tsetse catch, based upon our earlier work and a large set of pre-existing experimental data. We reconstructed those expectations for the current study by fitting the following model to the data reported in that publication [4], generating probability distributions for the relevant parameters:

$$\begin{array}{l} total\;catch∼Poisson(\lambda )\\ log(\lambda )=\alpha +\log\left(10\right)+{z\alpha }_{rep[i]}\times \sigma_{rep}+{z\alpha }_{expt[i]}\times \sigma_{expt}+\beta_{R7p}\times R7p+\beta_{R7y}\times R7y+\beta_{R8y}\times R8y\\ \alpha ∼Normal(0,10)\\ \beta_{R7p}∼Normal(0,10)\\ \beta_{R7y}∼Normal(0,10)\\ \beta_{R8y}∼Normal(0,10)\\ {z\alpha }_{rep}∼Normal(0,1)\\ {z\alpha }_{expt}∼Normal(0,1)\\ \sigma_{rep}∼HalfNormal(0,1)\\ \sigma_{expt}∼HalfNormal(0,1) \end{array}$$

The predictors R7p, R7y, and R8y are the three photoreceptor signals found to be most influential upon tsetse attraction in an earlier analysis of these data [8–10] (here we have used generic fly photoreceptor sensitivity functions, as recorded in *Musca* and *Calliphora* [12]). Since the data available in the prior publication were totals across 10 sampling days per experiment [4], an offset was included to transform predictions to a daily scale and a random intercept for experiment (rather than site and day) was modelled. The priors here are regularising only, as in the models described above.

The posterior distributions of the photoreceptor signal coefficients from this analysis (with slightly inflated standard deviations) were then used as priors for a similar analysis of the data from our two experiments in Kenya combined. Photoreceptor excitations for the fabrics we tested were computed from their measured reflectance spectra (Fig 1) using the calculator provided with our earlier work [9]. For simplicity, we refer the reader to that work for a detailed explanation of the calculations involved. The pertinent aspects of this analysis are as follows, with the omitted priors remaining in the regularising forms described above:

$$\begin{array}{l} total\;catch∼Poisson(\lambda )\\ log(\lambda )=\alpha +{z\alpha }_{rep[i]}\times \sigma_{rep}+{z\alpha }_{day[i]}\times \sigma_{day}+{z\alpha }_{site[i]}\times \sigma_{site}+\beta_{R7p}\times R7p+\beta_{R7y}\times R7y+\beta_{R8y}\times R8y\\ \beta_{R7p}∼Normal(-2.1,0.5)\\ \beta_{R7y}∼Normal(2.1,0.5)\\ \beta_{R8y}∼Normal(-1.2,0.5) \end{array}$$

The posterior distributions of the photoreceptor signal coefficients from this analysis in turn formed the priors for an analysis of the data from our experiment in Uganda using a model of the above form, and a zero-inflated model. The pertinent modifications for the zero-inflated model were as follows, and the stated priors were identical in the non-zero-inflated model:

$$\begin{array}{l} total\;catch∼ZIPoisson(p,\lambda )\\ logit(p)=\alpha_{p}\\ log(\lambda )=\alpha +{z\alpha }_{rep[i]}\times \sigma_{rep}+{z\alpha }_{day[i]}\times \sigma_{day}+{z\alpha }_{site[i]}\times \sigma_{site}+\beta_{R7p}\times R7p+\beta_{R7y}\times R7y+\beta_{R8y}\times R8y\\ \beta_{R7p}∼Normal(-2.0,0.4)\\ \beta_{R7y}∼Normal(2.2,0.5)\\ \beta_{R8y}∼Normal(-0.9,0.3) \end{array}$$

In this way, our analyses estimated the plausible distributions of model coefficients that are consistent with all of our experimental data, as well as a larger set of data for the same species published prior to our study [4].

#### Evaluating model effects through future catch predictions

The above models estimate posterior probability distributions for each coefficient in the linear model that are consistent with the specified priors and the data under analysis. These posterior distributions explicitly represent uncertainty in model parameters. To evaluate each model, we simulated 10,000 new catch replicates for the relevant target set by sampling randomly from the posterior distributions of the model’s coefficients. In addition, new z-scores for day, site, and replicate were randomly generated for each, to simulate variation beyond the days, sites, and replicates sampled in our experiments. Using these samples, we computed expected tsetse catches, λ, for each target. First, we evaluated the likelihood of target colour effects by computing expected catches for the average catch replicate, that is, with the z-scores for each simulated catch replicate set to zero so that variability in catches across replicates did not contribute to predictions. Next, we evaluated the likelihood of observing a catch difference between targets at any given replicate by computing expected catches using the randomly generated z-scores for each replicate to incorporate the expected variability in catches. Based on these simulations, we compared expected catches across pairs of targets at the same simulated site and on the same simulated day, evaluating the probability that the expected catch of a given target would exceed that of another. In this way, our predictions describe the probable effects of target colour on the average catch replicate, as well as the probability of one target outperforming another in any given replicate, given the expected variability in catches around the average. The reader should be aware that sampling variation across new intercepts for day, site, and replicate had considerable effect on the shape of the expected catch density functions we present in results. However, median predicted catches and performance comparisons for different targets were consistent.

Since our analysis based on photoreceptor signals was intended to generalise patterns across experiments and the two families of improved fabrics, rather than making predictions for the actual targets tested, we constructed these for typical blue, ‘average Vestergaard’, and ‘average violet’ fabrics. The reflectance spectra for the latter two hypothetical fabrics were generated by averaging across those of the several fabrics within that family shown in Fig 1.

## Results

### An analysis of Tiny Target performance without informative priors

#### Tiny Targets in Kenya

**Fig 2:**
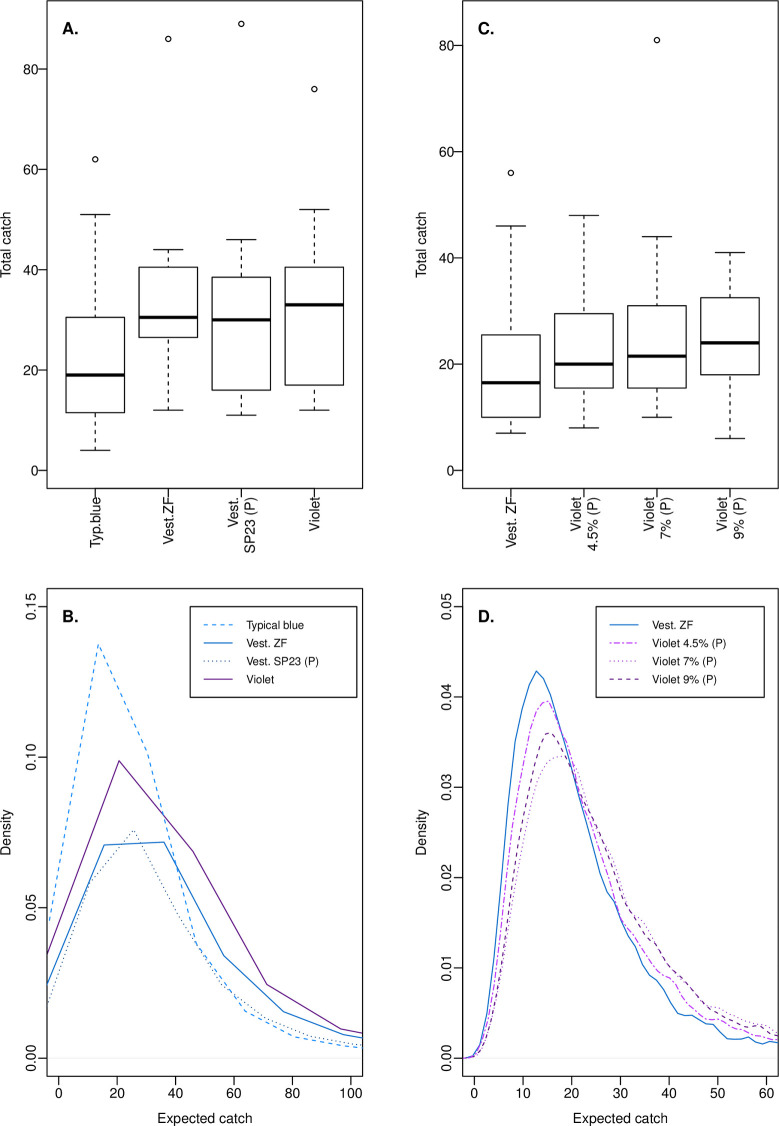
Recorded and posterior predicted *G*. *f*. *fuscipes* catches based on experiments in Kenya. (A) The distribution of recorded tsetse catches for the four target fabrics tested in experiment 1. (B) Probability density functions for 10,000 expected catches for the targets tested in experiment 1, computed using the posterior probabilities of model coefficients from a Bayesian model of the data in panel A. (C) The distribution of recorded tsetse catches across the four target fabrics tested in experiment 2. (D) Probability density functions for 10,000 expected catches for each target tested in experiment 2, computed using the posterior probabilities of model coefficients from a Bayesian model of the data in panel C. Boxes in panels A and C indicate the 25^th^, 50^th^, and 75^th^ percentiles; whiskers extend to the most extreme value, or to 1.5 * IQR with more extreme values plotted as points. Sample sizes are 16 catch observations per target. In panels B and D, note that sampling variation across new intercepts for day, site, and replicate has considerable effect on the shape of the plotted functions, but not the medians and performance comparisons stated in text.

Our first experiment compared catches at a typical blue polyester target comparable to those tested by [4], and targets using the putatively improved Vestergaard blue and violet fabrics (Fig 2A). A statistical model of these catches revealed that the plausible distribution of the coefficient for the typical blue polyester target had its probability mass below zero, indicating high certainty of lesser performance than the Vest. ZF target (Table 1). Mean coefficients for the violet and Vest. SP23 prototype were slightly negative and their probability masses straddled zero, indicating uncertainty in performance relative to Vest. ZF, but suggesting that these targets tended to perform slightly less well (Table 1). Also evident in this model was relatively high catch variability across days and sites (σ_day_ and σ_site_), and additional catch variability across the individual catch replicates (σ_rep_) that was not explained by the other predictors in the model (Table 1).

**Table 1:** A summary of posterior distributions for the coefficients estimated by analysis of experiment 1 data.

	Mean	SD	5.50% HPDI	94.50% HPDI	N_effective_	$\hat{R}$
α	3.39	0.36	2.85	3.92	8721	1
β_Typ.Blue_	-0.42	0.12	-0.62	-0.22	15125	1
β_Vest.SP23(P)_	-0.08	0.12	-0.27	0.12	13876	1
β_Violet_	-0.04	0.12	-0.23	0.15	14710	1
σ_day_	0.32	0.09	0.20	0.47	9049	1
σ_site_	0.58	0.31	0.26	1.15	10821	1
σ_rep_	0.28	0.05	0.21	0.36	8759	1

SD = standard deviation; HPDI = highest posterior density interval; N_effective_ (= the effective number of samples) and $\hat{R}$ are convergence and efficiency diagnostics for Markov chains used in model fitting.

To evaluate these effects, we calculated the expected catch, λ, for 10,000 simulated target catch replicates that captured uncertainty in model coefficients, and variation across sites, days, and replicates (Table 2 and Fig 2B). Across these simulations, median expected catches for typical blue (19.7) were less than those for Vest. ZF (29.7), Vest. SP23 (27.5), and violet (28.5), and the distributions of expected catches for those putatively improved fabrics were similar (Fig 2B). To evaluate target colour effects independent of the estimated variability in catches between replicates, we compared expected catches for the average catch replicate for each target type at the same simulated site and on the same simulated day. From these comparisons it was deemed certain that the average expected catch of each putatively improved target would exceed that of typical blue (Table 2). Among pairs of the putatively improved target fabrics it was marginally more likely that Vest. ZF would have the greater expected catch (Table 2). We next expanded these predictions to incorporate variability in catches across replicates, wherein the probability of one putatively improved fabric having a higher expected catch than another at any given replicate was marginal, and the probability of one of these fabrics having a higher expected catch than typical blue was reduced to ca. 79–84% (Table 2, values in brackets).

**Table 2:** Future target performance predicted by analysis of experiment 1 data.

	Percentage of deployments in which expected catch exceeds. . .			
Target	(i) Typ. Blue	(ii) Vest. ZF	(iii) Vest. SP23 (P)	(iv) Violet
**Typ. Blue**		0% (16%)	0% (21%)	0% (18%)
**Vest. ZF**	100% (84%)		73% (58%)	64% (54%)
**Vest. SP23 (P)**	100% (79%)	27% (42%)		39% (46%)
**Violet**	100% (82%)	36% (46%)	61% (54%)	

Table shows the percentage of 10,000 simulated catches in which the expected catch (λ) of the target in the row heading exceeded that in the column heading for the average catch replicate. Values in brackets indicate the same comparison of catch predictions, but incorporate the additional variability in tsetse catches across replicates estimated by the model.

Our second experiment tested a number of violet fabric prototypes against the Vest. ZF fabric tested in our first experiment (Fig 2C). Among the violet fabrics, we expected that increasing dye concentrations would result in increased attractiveness to tsetse, based upon our previous analyses of fly photoreceptor signals [8,9]. Although produced using the same dye and base fabric as the violet previously tested, these were produced on a small scale, which resulted in the fabrics being more crinkled. Analysis of these data suggested that each violet target had a positive coefficient (Table 3). The distribution of plausible values for this coefficient was almost entirely positive for violet 7% and violet 9% (Table 3), both of which had very similar reflectance spectra to the violet tested in experiment 1 (Fig 1). Approaching one quarter of the probability mass for the violet 4.5% coefficient was below zero, indicating greater uncertainty in its effect (Table 3). There was, again, high variability in tsetse catches across days and sites, and additional variability across catch replicates that was not explained by other predictors in the model (Table 3).

**Table 3:** A summary of posterior distributions for the coefficients estimated by analysis of experiment 2 data.

	Mean	SD	5.50% HPDI	94.50% HPDI	N_effective_	$\hat{R}$
α	2.89	0.19	2.58	3.19	11470	1
β_Violet4.5%_	0.10	0.14	-0.12	0.32	16255	1
β_Violet7%_	0.25	0.14	0.04	0.47	15957	1
β_Violet9%_	0.21	0.14	-0.01	0.43	15828	1
σ_day_	0.32	0.09	0.19	0.48	8900	1
σ_site_	0.38	0.14	0.21	0.64	11920	1
σ_rep_	0.31	0.06	0.23	0.41	8078	1

SD = standard deviation; HPDI = highest posterior density interval; N_effective_ (= the effective number of samples) and $\hat{R}$ are convergence and efficiency diagnostics for Markov chains used in model fitting.

Across 10,000 simulated catch replicates for each target based on this model, the median expected catch for Vest. ZF was 17.9, and those for the violet fabrics were greater (violet 4.5% = 19.7; violet 7% = 22.9; violet 9% = 22.0) (Fig 2D). Considering the average catch replicate at the same simulated site and day, there was a 77–97% probability that the average expected catch across replicates of a violet prototype target would exceed that at a Vest. ZF target (Table 4), reversing the trend observed in analysis of our first experiment. This probability was greatest (>90%) for the 7% and 9% violet prototypes, which had similar reflectance spectra to the violet tested in our first experiment (see Fig 1), in agreement with our prior expectations. However, as for our first experiment, when the additional variability in catches across replicates was incorporated into these predictions, the probability of a greater expected catch at a violet prototype target than a Vest. ZF target in any given replicate at the same day and site was marginal (Table 4).

**Table 4:** Future target performance predicted by analysis of experiment 2 data.

	Percentage of deployments in which expected catch exceeds…			
Target	(i) Vest. ZF	(ii) Violet 4.5% (P)	(iii) Violet 7% (P)	(iv) Violet 9% (P)
**Vest. ZF**		23% (41%)	3% (29%)	7% (32%)
**Violet 4.5% (P)**	77% (59%)		13% (36%)	21% (40%)
**Violet 7% (P)**	97% (71%)	87% (64%)		62% (54%)
**Violet 9% (P)**	93% (68%)	79% (60%)	38% (46%)	

Table shows the percentage of 10,000 simulated catches in which the expected catch (λ) of the target in the row heading exceeded that in the column heading for the average catch replicate. Values in brackets indicate the same comparison of catch predictions, but incorporate the additional variability in tsetse catches across replicates estimated by the model.

#### Tiny Targets in Uganda

**Fig 3:**
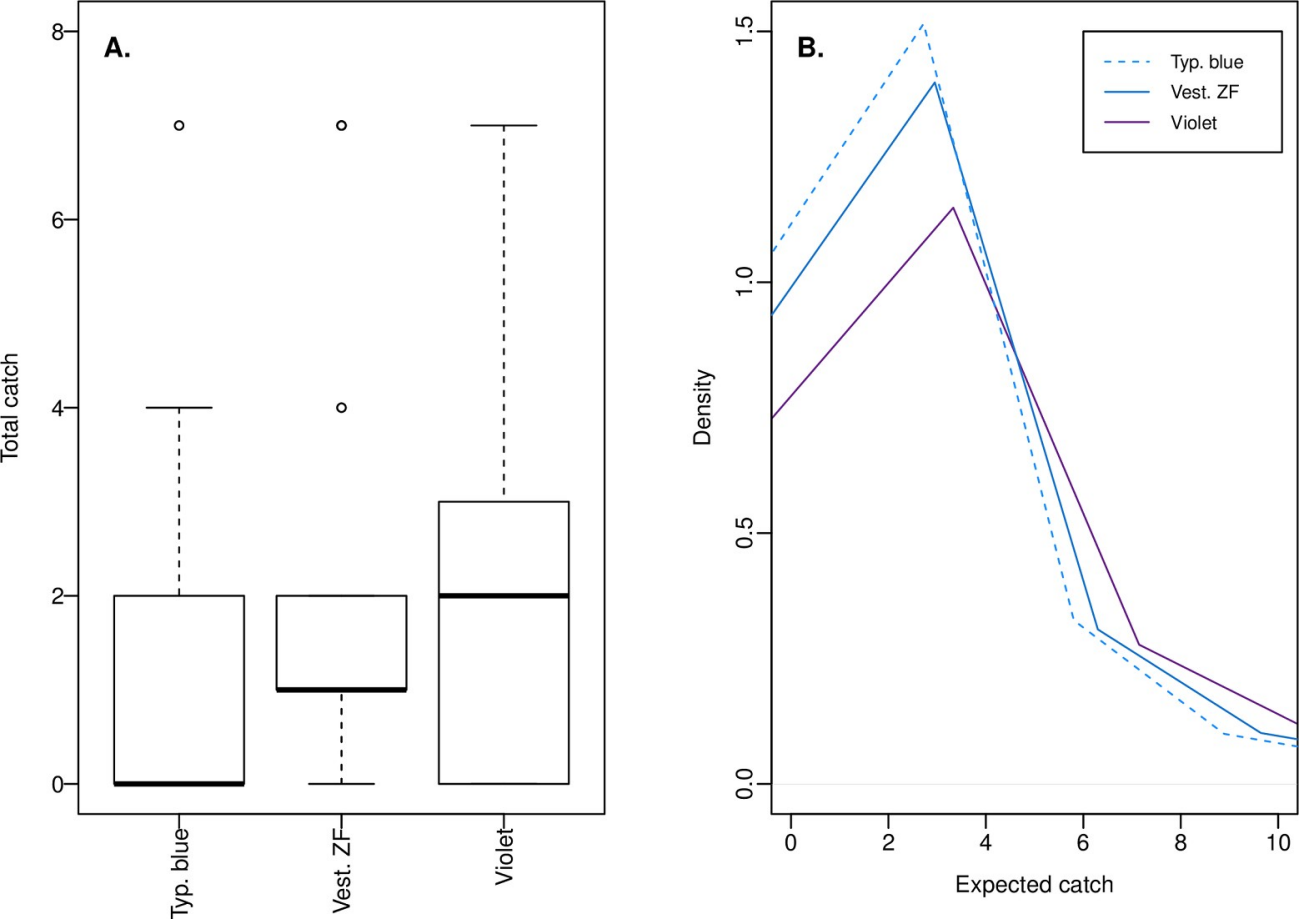
Recorded and posterior predicted *G*. *f*. *fuscipes* catches based on the experiment in Uganda. (A) The distribution of recorded tsetse catches across the three target fabrics tested. (B) Probability density functions for 10,000 expected catches for the same targets, computed using posterior probabilities of model coefficients from a Bayesian model of catches in panel A. Predictions are from the zero-inflated model and exclude zero catches modelled to result from a binary logistic process independent of target type. Boxes in panel A indicate the 25^th^, 50^th^, and 75^th^ percentiles; whiskers extend to the most extreme value, or to 1.5 * IQR with more extreme values plotted as points. Sample sizes are 17 catch observations for Vest. ZF and violet, 16 for typical blue. In panel B, note that sampling variation across new intercepts for day, site, and replicate has considerable effect on the shape of the plotted functions, but not the medians and performance comparisons stated in text.

Catches of tsetse during our third experiment in Uganda were generally low and often zero (Fig 3A), so we applied both an over-dispersed Poisson model, and a zero-inflated version of this model to account for replicates in which there may have been no tsetse in the vicinity of a target (structural zeroes). In the zero-inflated model, structural zero catches were predicted in about 22% of target replicates. For both models, in common with experiments in Kenya, the coefficient for the violet target effect was positive and that for the typical blue target negative, though both probability masses straddled zero indicating some uncertainty in the magnitude and direction of these effects (Table 5). Both models evidenced high variability in tsetse catches across days and sites (Table 5). Although the zero-inflated model performed less well as judged by information criteria (difference in WAIC = +6.9, standard error of difference = 3.1), it contained less unexplained variability across replicates (σ_rep_) closer to that observed in our experiments in Kenya (Table 5).

**Table 5:** A summary of posterior distributions for the coefficients estimated by analysis of experiment 3 data using (A) over-dispersed Poisson, and (B) zero-inflated over-dispersed Poisson models.

	Mean	SD	5.50% HPDI	94.50% HPDI	N_effective_	$\hat{R}$
**(A)**						
α	0.15	0.63	-0.84	1.09	11715	1
β_Violet_	0.07	0.40	-0.56	0.69	17541	1
β_Typ.Blue_	-0.45	0.43	-1.15	0.21	17626	1
σ_day_	0.49	0.29	0.06	0.99	5642	1
σ_site_	0.82	0.44	0.28	1.63	14814	1
σ_rep_	0.69	0.28	0.21	1.13	4981	1
**(B)**						
α_p_	-1.32	0.53	-2.22	-0.57	18872	1
α	0.41	0.59	-0.54	1.28	10362	1
β_Violet_	0.22	0.34	-0.33	0.77	18902	1
β_Typ.Blue_	-0.14	0.40	-0.79	0.49	18987	1
σ_day_	0.37	0.25	0.04	0.82	6306	1
σ_site_	0.79	0.43	0.27	1.57	16324	1
σ_rep_	0.41	0.25	0.05	0.83	5369	1

SD = standard deviation; HPDI = highest posterior density interval; N_effective_ (= the effective number of samples) and $\hat{R}$ are convergence and efficiency diagnostics for Markov chains used in model fitting.

Median expected catches across 10,000 simulated catch replicates for each target were 0.8 for typical blue, 1.2 for Vest. ZF, and 1.3 for violet based on the non-zero-inflated model, and 1.4 for typical blue, 1.6 for Vest. ZF, and 2.0 for violet based on the zero-inflated model excluding structural zeroes. Based on the average simulated catch replicate at the same simulated site and day, the non-zero-inflated model predicted a ≥86% probability of a higher expected catch at violet or Vest. ZF targets than at typical blue, but the difference between violet and Vest. ZF targets was marginal (Table 6). The zero-inflated model predicted an 81% probability of a higher expected catch at the violet than the typical blue target, but only a 64% probability of a higher expected catch at Vest. ZF than typical blue (Table 6 and Fig 3B). In addition, there was a 75% probability of a greater expected catch at violet than Vest. ZF (Table 6). However, as with previous simulations, when between replicate catch variability was incorporated into these predictions, only the probability of violet having a higher expected catch than typical blue at any given target catch replicate remained at ≥70% (Table 6).

**Table 6:** Future target performance predicted by analysis of experiment 3 data using (A) over-dispersed Poisson, and (B) zero-inflated over-dispersed Poisson models.

	Percentage of deployments in which expected catch exceeds …		
Target	(i) Typ. Blue	(ii) Vest. ZF	(iii) Violet
**(A)**			
**Typ. Blue**		14% (31%)	10% (30%)
**Vest. ZF**	86% (69%)		43% (48%)
**Violet**	90% (70%)	57% (52%)	
**(B)**			
**Typ. Blue**		36% (41%)	19% (30%)
**Vest. ZF**	64% (59%)		25% (36%)
**Violet**	81% (70%)	75% (64%)	

Table shows the percentage of 10,000 simulated catches in which the expected catch (λ) of the target in the row heading exceeded that in the column heading for the average catch replicate. Values in brackets indicate the same comparison of catch predictions, but incorporate the additional variability in tsetse catches across replicates estimated by the model.

### A fly photoreceptor signal-based analysis using informative priors

The above analyses by experiment suggest trends in target performance, but these were obscured by high variability in tsetse catches, and were sometimes inconsistent between experiments. Those analyses used regularising priors, which means that they took no account of prior expectations of target performance, despite the fact that such expectations were overtly drawn from earlier work and informed the design of the targets tested [8–10]. We next took advantage of the ability of Bayesian analyses to incorporate these prior expectations and consolidate patterns across experiments.

**Fig 4:**
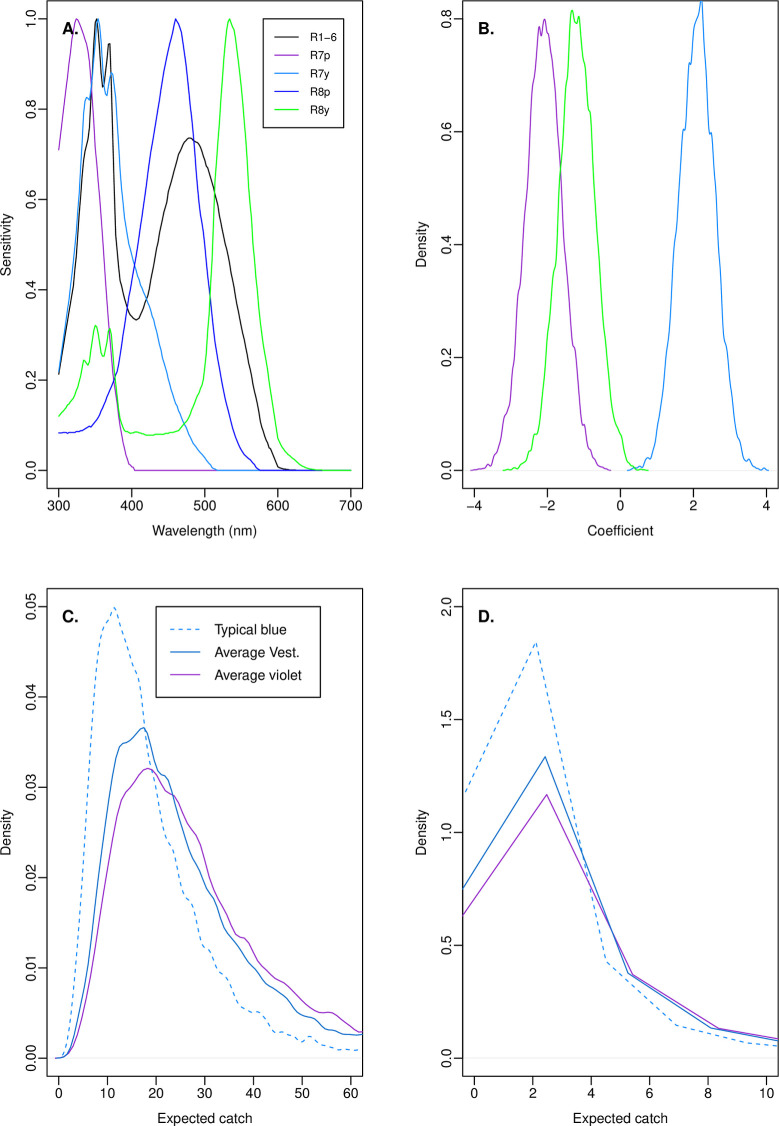
Bayesian analysis of *G*. *f*. *fuscipes* catches using informative priors established from published data. (A) Flies possess five classes of photoreceptor with characteristic spectral sensitivities [12], and the responses of these photoreceptors to target fabrics provide the only inputs to visually guided behaviour. The responses of these photoreceptors to each fabric can be calculated from the fabric’s reflectance spectrum and used as predictors of attraction in statistical models of tsetse catches [8,9]. (B) Prior probabilities for coefficients relating calculated photoreceptor responses to tsetse catches, based on analysis of data published in [4]. Only the three photoreceptors deemed most influential in earlier work have been considered [8,9]. Plot shows random samples with the means and standard deviations specified in methods. (C) Probability density functions for 10,000 expected catches computed for typical blue, ‘average Vestergaard’, and ‘average violet’ targets based upon the posterior probabilities of model coefficients generated from the priors in panel B, and recorded catches from experiments 1 and 2 in Kenya. (D) Probability density functions for 10,000 expected catches computed for typical blue, ‘average Vestergaard’, and ‘average violet’ targets based upon the posterior probabilities of coefficients for the model described in panel C, and recorded catches for experiment 3 conducted in Uganda. Predictions are from the zero-inflated model and exclude zero catches modelled to result from a binary logistic process independent of target type. Data in panel A are as used in [9] and based on [12]. In panels C and D, note that sampling variation across new intercepts for day, site, and replicate has considerable effect on the shape of the plotted functions, but not the medians and performance comparisons stated in text.

Flies possess five spectral classes of photoreceptor that provide the inputs to their visually guided behaviour (Fig 4A). Relationships between calculated signals for these photoreceptors, and numbers of tsetse attracted to targets in previous experiments, were the basis for development of the violet fabrics tested in this work [8–10]. We therefore began by re-analysing the total catches reported in a pre-existing dataset of *G*. *f*. *fuscipes* catches at coloured Tiny Targets [4], to reconstruct probability distributions for the coefficients of calculated photoreceptor signals that could provide informative priors for use in the analysis of our new data (Table 7).

**Table 7:** A summary of posterior distributions for the coefficients estimated by analysis of the data collected by [4].

	Mean	SD	5.50% HPDI	94.50% HPDI	N_effective_	$\hat{R}$
α	3.65	0.14	3.43	3.88	8835	1
β_R7p_	-2.10	0.19	-2.41	-1.79	9639	1
β_R7y_	2.09	0.30	1.60	2.57	8571	1
β_R8y_	-1.21	0.25	-1.61	-0.81	9185	1
σ_expt_	0.19	0.05	0.13	0.28	6993	1
σ_rep_	0.17	0.02	0.14	0.20	7357	1

SD = standard deviation; HPDI = highest posterior density interval; N_effective_ (= the effective number of samples) and $\hat{R}$ are convergence and efficiency diagnostics for Markov chains used in model fitting.

The posterior probability distributions of the photoreceptor signal coefficients from this analysis informed the prior expectations for a single analysis incorporating both of our Kenya experiments (Fig 4B). This analysis produced new posterior probability distributions for these coefficients consistent with both our data and information in the specified priors (Table 8). We used this model to simulate 10,000 deployments of a typical blue, ‘average Vestergaard blue’, and ‘average violet’ Tiny Target (the latter two calculated by averaging the reflectance spectra of the actual fabrics tested), incorporating the modelled variability across sites, days, and replicates (Fig 4C). Again, median expected catches for average violet (24.6) and average Vestergaard blue (21.9) exceeded that for typical blue (15.7). From these simulations, expected catches for the average replicate at average violet and average Vestergaard blue targets were deemed certain to exceed those at typical blue, and it was highly probable that that for average violet would exceed that for average Vestergaard blue (Table 9). However, when between-replicate catch variability was incorporated into these simulations, the advantage for violet over Vestergaard blue was diluted and the probability that putatively improved fabrics would outperform typical blue fell to 79–86% (Table 9).

**Table 8:** A summary of posterior distributions for the coefficients estimated by analysis of new data collected in Kenya, incorporating priors informed by analysis of previously published data [4].

	Mean	SD	5.50% HPDI	94.50% HPDI	N_effective_	$\hat{R}$
α	2.96	0.34	2.41	3.51	14334	1
β_R7p_	-1.97	0.37	-2.57	-1.38	22614	1
β_R7y_	2.22	0.47	1.47	2.97	21834	1
β_R8y_	-0.89	0.25	-1.30	-0.49	18578	1
σ_day_	0.32	0.06	0.23	0.41	8699	1
σ_site_	0.40	0.11	0.26	0.60	9421	1
σ_rep_	0.28	0.03	0.23	0.34	10319	1

SD = standard deviation; HPDI = highest posterior density interval; N_effective_ (= the effective number of samples) and $\hat{R}$ are convergence and efficiency diagnostics for Markov chains used in model fitting.

**Table 9:** Future target performance predicted by analysis of new data collected in Kenya, incorporating priors informed by analysis of previously published data [4].

	Percentage of deployments in which expected catch exceeds …		
Target	(i) Typ. Blue	(ii) ‘Ave. Vest.’	(iii) ‘Ave. violet’
**Typ. Blue**		0% (21%)	0% (14%)
**‘Average Vest.’**	100% (79%)		4% (39%)
**‘Average violet’**	100% (86%)	96% (61%)	

Table shows the percentage of 10,000 simulated catches in which the expected catch (λ) of the target in the row heading exceeded that in the column heading for the average catch replicate. Values in brackets indicate the same comparison of catch predictions, but incorporate the additional variability in tsetse catches across replicates estimated by the model.

We next used the posterior probability distributions from this analysis to provide revised prior expectations for an analysis of our Uganda experiment, consolidating all of our new data with that published in the pre-existing study [4]. As in our initial analysis of that experiment, we modelled these data both with and without zero-inflation that might have resulted from there being no tsetse in the vicinity of any given target replicate. The results of this analysis evidenced a weakening of the coefficients indicated in the priors, but the two models were comparable (Table 10). We again used these models to simulate 10,000 deployments of a typical blue, average Vestergaard blue, and average violet Tiny Target, wherein median expected catches were 0.8 for typical blue, 1.1 for average Vestergaard blue, and 1.3 for average violet, based upon the non-zero-inflated model, and 1.2 for typical blue, 1.6 for average Vestergaard blue, and 1.9 for average violet based on the zero-inflated model and excluding structural zeroes (Fig 4D). Predictions of relative target performance based upon these simulations were similar to those in the previous analysis and across both statistical models (Table 11). For the average catch replicate at the same simulated site and day, both average Vestergaard blue and average violet targets were certain to yield higher expected catches than typical blue, and it was moderately likely that average violet would yield a higher expected catch than average Vestergaard blue (Table 11). However, once between-replicate variability was incorporated into these predictions, the performance advantage for average violet over average Vestergaard blue was marginal, and the probability that either would outperform typical blue fell to 69–82% (Table 11).

**Table 10:** A summary of posterior distributions for the coefficients estimated by analysis of new data collected in Uganda, incorporating priors from the analysis of data collected in Kenya that itself used informative priors. Coefficients are displayed for (A) over-dispersed Poisson, and (B) zero-inflated over-dispersed Poisson models.

	Mean	SD	5.50% HPDI	94.50% HPDI	N_effective_	$\hat{R}$
**(A)**						
α	0.03	0.71	-1.11	1.14	12101	1
β_R7p_	-2.01	0.39	-2.62	-1.39	37770	1
β_R7y_	2.20	0.49	1.42	2.98	32072	1
β_R8y_	-0.90	0.29	-1.36	-0.44	42201	1
σ_day_	0.51	0.30	0.07	1.00	4602	1
σ_site_	0.82	0.43	0.29	1.62	15675	1
σ_rep_	0.60	0.27	0.15	1.03	3965	1
**(B)**						
α_p_	-1.36	0.53	-2.26	-0.59	17950	1
α	0.37	0.71	-0.73	1.47	11457	1
β_R7p_	-1.95	0.39	-2.57	-1.33	33030	1
β_R7y_	2.21	0.49	1.43	2.99	26294	1
β_R8y_	-0.90	0.28	-1.35	-0.45	33198	1
σ_day_	0.37	0.25	0.04	0.81	6320	1
σ_site_	0.81	0.43	0.29	1.60	14102	1
σ_rep_	0.37	0.23	0.04	0.77	6266	1

SD = standard deviation; HPDI = highest posterior density interval; N_effective_ (= the effective number of samples) and $\hat{R}$ are convergence and efficiency diagnostics for Markov chains used in model fitting.

**Table 11:** Future target performance estimated by analysis of new data collected in Uganda, incorporating priors from the analysis of data collected in Kenya that itself used informative priors. Predictions are displayed for (A) over-dispersed Poisson, and (B) zero-inflated over-dispersed Poisson models.

	Percentage of deployments in which expected catch exceeds …		
Target	(i) Typ. Blue	(ii) ‘Ave. Vest.’	(iii) ‘Ave. violet’
**(A)**			
**Typ. Blue**		0% (32%)	0% (27%)
**‘Average Vest.’**	100% (69%)		12% (42%)
**‘Average violet’**	100% (73%)	88% (58%)	
**(B)**			
**Typ. Blue**		0% (23%)	0% (18%)
**‘Average Vest.’**	100% (77%)		12% (38%)
**‘Average violet’**	100% (82%)	88% (62%)	

Table shows the percentage of 10,000 simulated catches in which the expected catch (λ) of the target in the row heading exceeded that in the column heading for the average catch replicate. Values in brackets indicate the same comparison of catch predictions, but incorporate the additional variability in tsetse catches across replicates estimated by the model.

## Discussion

In this work we evaluated the performance of putatively improved coloured fabrics for the Tiny Targets used to control riverine tsetse, based upon new experimental trials in Kenya and Uganda. Using a Bayesian statistical approach, we found strong evidence that expected catches for future deployments of violet and Vestergaard blue targets were likely to exceed those for typical blue polyester targets. Among these improved fabrics, our analyses suggest that expected catches for average violet target replicates were moderately likely to exceed those for Vestergaard blue, with a small difference in median expected catches of ca. +15% (range across analyses: -4% to +28%). However, because variability in tsetse catches between replicates is large, the probability that the expected catch for any given violet target replicate would exceed that for a Vestergaard blue equivalent was marginal (46–71%).

Recently, we conducted tests of the same fabrics against savannah tsetse, using the large (2 m wide x 1 m high) targets recommended for their control [10]. We found that violet targets often caught significantly more tsetse than typical blue polyester and black cotton standard ones, whilst Vest. ZF blue targets did not attain significantly higher catches than black cotton [10]. Thus, although no direct test was conducted, these data suggest that violet targets were likely more attractive to these flies than Vest. ZF ones. Previous studies of tsetse attracted to traps and targets have identified similar colour features as important in enhancing catches of both savannah and riverine species [4,5,7,8,13]. Therefore, we anticipated that the relative attractiveness of these fabrics to riverine tsetse would follow the trends for savannah species. We evaluated this by making future catch predictions for the average catch replicate, ignoring variability between replicates and focussing on uncertainty in target colour effects only. In the majority of these analyses, the greater attractiveness of violet and Vest. ZF fabrics over a typical blue like those that have been tested previously was very highly probable, though such an advantage was not evident for Vest. ZF in the zero-inflated analysis of our third experiment. In most analyses, expected catches for the average replicate deployment of violet fabrics were moderately likely to exceed those for Vest. ZF, but the difference in median expected catches was small and an opposite trend was indicated by our individual analysis of our first experiment. Further, among our prototype violet fabrics in our second experiment, those with higher dye concentrations were likely to achieve higher expected catches than that with a lower dye concentration, aligning with our prior expectations from analyses of fly photoreceptor signals. Thus, our data are broadly consistent with a similar ordering of colour preference for *G*. *f*. *fuscipes* as was seen for savannah species, albeit that the magnitude of expected catch difference between violet and Vestergaard blue targets was small.

Our analysis of expected catches for average target replicates did not account for the high variability in catches between replicates. When this variability was incorporated into predictions, the expected catch at a given violet or Vest. ZF target replicate retained a moderate probability of exceeding that at a typical blue replicate on the same day and at the same site. Thus, even given the high variability in tsetse catches, these improved fabrics appear to have a relatively clear performance advantage over typical blue. However, the probability that the expected catch for a given violet target replicate would exceed that for a Vest. ZF equivalent settled closer to random chance. In this case, the high variability in catches swamped the relatively small difference between median expected catches. Although we maintain that Bayesian statistics provide the most useful way to interrogate our data, and allowed that understanding to be consolidated across multiple experiments, we note that separate analyses of each experiment using frequentist statistical methods aligned with this finding: the catches of violet and Vest. ZF targets never differed significantly, but these catches did significantly exceed those of typical blue in our first experiment (though not in our third experiment where extremely low numbers of flies were caught and the median catches for all targets were ≤ 2) (See S1 Statistics). Thus, the high variability in expected tsetse catches appears to obscure the colour effects and contributes to the discrepancy in findings versus those for savannah species [10]. We suggest that this high variability may be a feature of Tiny Target deployments against riverine species, since those species are less discriminating than savannah tsetse over targets as indicated by their relative indifference to target size [14]. This may be because riverine tsetse are less discriminating over potential hosts due to the way in which their search behaviour is constrained by habitat [14,15]. The high variability in riverine tsetse catches at Tiny Targets may have important ramifications for the optimisation of these devices, since it appears to impose a limit on the degree to which target performance can be improved.

It has also been proposed that divergence in opsin sequences across tsetse species may lead to differences in phototoreceptor sensitivity, and thus, colour-guided behaviour [16]. Conceivably, this could contribute to the weaker trends in relative target attractiveness seen for riverine tsetse in this study, versus those for savannah tsetse in previous work [10]. However, whether or not the quantified divergence in opsin sequences leads to meaningful differences in photoreceptor spectral sensitivity remains to be seen. Divergence in opsin sequences between tsetse species was much less than that between those species and *Musca domestica* [16], yet electrophysiological study found little difference between the spectral sensitivities of four of the five main photoreceptor types between *Glossina morsitans* and *M*. *domestica* [17]. However, the largest sequence divergence occurs for opsin Rh5 which is expressed in the blue-sensitive R8p photoreceptor class (see Fig 4A) [16], and that photoreceptor type has never been successfully recorded from in tsetse [17]. For this reason, a *Musca*-like R8p sensitivity function was used in previous models explaining tsetse catches based on photoreceptor signals, where its response proved not to be an important predictor of attraction [8,9]. Therefore, it remains to be seen whether the spectral sensitivity of the R8p photoreceptor varies across tsetse species, and between those species and *M*. *domestica*, and whether that photoreceptor makes an important contribution to attraction. Electrophysiological and field experimentation, and models of tsetse behaviour based upon photoreceptor signals, are required to address this problem.

In developing the fabrics tested in this work we focussed on fabric colour properties, quantified from a fly’s eye view using calculated fly photoreceptor signals [8–10]. Many biting flies are most attracted to darker objects and those reflecting blue wavelengths, and least attracted to objects that reflect UV and green wavelengths [4,5,7,18,19]. This is likely to be because, although blue objects are rare in the natural environment, they activate the same photoreceptor mechanisms that would normally discriminate the reflectance spectra of potential hosts from a background of green leaves [8,13,18]. But in addition to colour, the shiny surface texture of modern synthetic fabrics may also influence the attraction of biting flies [20–22]. For tabanids, polarised reflectance from shiny surfaces is important in the location of water bodies for egg laying and mating [22], and could aid in the discrimination of dark, shiny-furred hosts from their background [23]. Within the tabanid visual system, colour and polarisation sensation appear to be confounded because a blue-sensitive photoreceptor (analogous to R8p; see. Fig 4A), and a UV-sensitive photoreceptor (analogous to R7p) also differ in the plane of polarised light to which they are most sensitive [24]. As such, both colour and polarisation features might activate the same photoreceptor opponent signals, leading to the suggestion that the blue preference of these flies may be a by-product of this polarotactic mechanism [24]. Tsetse differ markedly to tabanids in that the high surface shininess of synthetic fabrics actually decreases catches [20,21]; and although some tsetse photoreceptors that contribute to colour vision are sensitive to the plane of polarised light [17], there is not yet evidence for opposing preferences among opponent pairs as was found for tabanids. Our violet and typical blue fabrics used the same base polyester as a deliberate control for surface texture, and the observed catch differences between these here, and for savannah tsetse [10], must be attributable to their colour properties. However, the Vestergaard blue fabrics had a noticeably more coarse and less shiny surface, and that may have enhanced their relative attractiveness to tsetse and obscured the expected advantage for violet fabrics (c.f. [20]). In support of this, our unset and relatively more crinkled violet 7% and 9% prototypes achieved the greatest performance increments over Vest. ZF, whilst our set violet fabric with a near identical reflectance spectrum performed more similarly to Vest. ZF. It is possible that violet dyes applied to the more textured Vestergaard base polyester would combine the advantages of both fabrics.

Our data provide evidence that improved polyester fabrics have indeed increased Tiny Target performance against riverine tsetse versus typical blues like those tested in earlier work [4]. The available evidence suggests that violet fabrics may be more attractive to both riverine and savannah tsetse than Vest. ZF ones, but that the high variability in riverine tsetse catches at Tiny Targets means that the current fabrics are likely to perform similarly when deployed in this context. Thus, we do not advocate a switch from Vestergaard ZeroFly blue to our current violet fabric for the sole purpose of improving Tiny Target catches. However, should a general purpose fabric for the effective control of both riverine and savannah species using both large and Tiny Target configurations be sought, we suggest that violet is the best overall choice. Finally, we caution practitioners and researchers that not all blue or violet materials are the same from a fly’s point of view, and that choices should be made based on reflectance spectra or fly-specific colour metrics, rather than colour appearance to a human observer [8,9].

## Supporting information

S1 Statistics Additional analyses for comparison to those reported in text. (DOCX)

S1 Dataset The complete set of data analysed in this study. (XLSX)

S1 R code R code that implements part 1 of the analysis described in text. (R)

S2 R code R code that implements part 2 of the analysis described in text. (R)

## References

[1] Solano P, Torr SJ, Lehane MJ. Is vector control needed to eliminate gambiense human African trypanosomiasis? Front Cell Infect Microbiol. 2013;3:33. doi: 10.3389/fcimb.2013.00033 23914350 PMC3728477

[2] Lindh JM, Torr SJ, Vale GA, Lehane MJ. Improving the cost-effectiveness of artificial visual baits for controlling the tsetse fly *Glossina fuscipes fuscipes*. PLoS Negl Trop Dis. 2009;3(7):e474. doi: 10.1371/journal.pntd.0000474 19582138 PMC2699553

[3] Esterhuizen J, Rayaisse JB, Tirados I, Mpiana S, Solano P, Vale GA, et al. Improving the cost-effectiveness of visual devices for the control of riverine tsetse flies, the major vectors of human African trypanosomiasis. PLoS Negl Trop Dis. 2011;5(8):e1257. doi: 10.1371/journal.pntd.0001257 21829743 PMC3149014

[4] Lindh JM, Goswami P, Blackburn R, Arnold SEJ, Vale GA, Lehane MJ, et al. Optimizing the colour and fabric of targets for the control of the tsetse fly *Glossina fuscipes fuscipes*. PLoS Negl Trop Dis. 2012;6(5):e1661. doi: 10.1371/journal.pntd.0001661 22666511 PMC3362611

[5] Green CH. The effect of colour on trap- and screen-oriented responses in *Glossina palpalis palpalis* (Robineau-Desvoidy) (Diptera: Glossinidae). Bull Ent Res. 1988;78:591–604.

[6] Green CH. Bait methods for tsetse fly control. Adv Parasitol. 1994;34:229–91. doi: 10.1016/s0065-308x(08)60140-2 7976751

[7] Green CH, Flint S. An analysis of colour effects in the performance of the F2 trap against *Glossina pallidipes* Austen and *G. morsitans morsitans* Westwood (Diptera: Glossinidae). Bull Ent Res. 1986;76:409–18.

[8] Santer RD. A colour opponent model that explains tsetse fly attraction to visual baits and can be used to investigate more efficacious bait materials. PLoS Negl Trop Dis. 2014;8(12):e3360. doi: 10.1371/journal.pntd.0003360 25473844 PMC4256293

[9] Santer RD. Developing photoreceptor-based models of visual attraction in riverine tsetse, for use in the engineering of more-attractive polyester fabrics for control devices. PLoS Negl Trop Dis. 2017;11(3):e0005448. doi: 10.1371/journal.pntd.0005448 28306721 PMC5371378

[10] Santer RD, Vale GA, Tsikire D, Torr SJ. Optimising targets for tsetse control: Taking a fly’s-eye-view to improve the colour of synthetic fabrics. PLoS Negl Trop Dis. 2019;13(12):e0007905. doi: 10.1371/journal.pntd.0007905 31830039 PMC6907749

[11] McElreath R. Statistical rethinking: A Bayesian course with examples in R and Stan. Boca Raton, FL: CRC Press; 2016.

[12] Hardie RC. The photoreceptor array of the dipteran retina. Trends Neurosci. 1986;9:419–23.

[13] Santer RD. A receptor-based explanation for tsetse fly catch distribution between coloured cloth panels and flanking nets. PLoS Negl Trop Dis. 2015;9(10):e0004121. doi: 10.1371/journal.pntd.0004121 26474406 PMC4608566

[14] Torr SJ, Vale GA. Know your foe: lessons from the analysis of tsetse fly behaviour. Trends Parasitol. 2015;31(3):95–9. doi: 10.1016/j.pt.2014.12.010 25599585

[15] Vale GA, Hargrove JW, Solano P, Courtin F, Rayaisse JB, Lehane MJ, et al. Explaining the host-finding behavior of blood-sucking insects: Computerized simulation of the effects of habitat geometry on tsetse fly movement. PLoS Negl Trop Dis. 2014;8(6):e2901. doi: 10.1371/journal.pntd.0002901 24921243 PMC4055578

[16] Attardo GM, Abd-Alla AMM, Acosta-Serrano A, Allen JE, Bateta R, Benoit JB, et al. Comparative genomic analysis of six Glossina genomes, vectors of African trypanosomes. Genome Biology. 2019;20(1):187. doi: 10.1186/s13059-019-1768-2 31477173 PMC6721284

[17] Hardie RC, Vogt K, Rudolph A. The compound eye of the tsetse fly (*Glossina morsitans morsitans* and *Glossina palpalis palpalis*). J Insect Physiol. 1989;35(5):423–31.

[18] Gibson G, Torr SJ. Visual and olfactory responses of haematophagous Diptera to host stimuli. Med Vet Entomol. 1999;13:2–23. doi: 10.1046/j.1365-2915.1999.00163.x 10194745

[19] Allan SA, Stoffolano JG Jr. The effects of hue and intensity on visual attraction of adult *Tabanus nigrovittatus* (Diptera: Tabanidae). J Med Entomol. 1986;23(1):83–91.

[20] Mihok S. The development of a multipurpose trap (the Nzi) for tsetse and other biting flies. Bull Ent Res. 2002;92:385–403. doi: 10.1079/BER2002186 12241564

[21] Mihok S, Carlson DA, Krafsur ES, Foil LD. Performance of the Nzi and other traps for biting flies in North America. Bull Ent Res. 2006;96(4):387–97. 16923207

[22] Horváth G, Majer J, Horváth L, Szivák I, Kriska G. Ventral polarization vision in tabanids: horseflies and deerflies (Diptera: Tabanidae) are attracted to horizontally polarized light. Naturwiss. 2008;95:1093–100. doi: 10.1007/s00114-008-0425-5 18685822

[23] Horváth G, Szörényi T, Pereszlényi Á, Gerics B, Hegedüs R, Barta A, et al. Why do horseflies need polarization vision for host detection? Polarization helps tabanid flies to select sunlit dark host animals from the dark patches of the visual environment. R Soc Open Sci. 2017;4:170735. doi: 10.1098/rsos.170735 29291065 PMC5717639

[24] Meglič A, Ilić M, Pirih P, Škorjanc A, Wehling MF, Kreft M, et al. Horsefly object-directed polarotaxis is mediated by a stochastically distributed ommatidial subtype in the ventral retina. Proc Nat Acad Sci USA. 2019;116(43):21843–53. doi: 10.1073/pnas.1910807116 31591223 PMC6815168

