# Supplementary material for: Evaluation of improved coloured targets to control riverine tsetse in East Africa: A Bayesian approach
Source: PLoS Negl Trop Dis. 2021 Jun 21;15(6):e0009463. doi: 10.1371/journal.pntd.0009463 (PMC8216509; doi:10.1371/journal.pntd.0009463)
Supplement: S1 Statistics — (DOCX) [file pntd.0009463.s001.docx]

**S1 Statistics**

**S1.1 Over-dispersed Poisson vs negative binomial models**

In our analyses, recorded catches varied more than expected for a Poisson distribution and were thus over-dispersed. We tackled this by fitting models which included a random intercept for each catch observation, explicitly modelling the extra variability in catches not accounted for by other parameters in the model [1]. An alternative solution would have been to fit negative binomial models. Tables A-J report the posterior distributions of coefficients generated using such negative binomial models, and relative catch predictions based on the mean expected catch, μ, generated from those models. The Rethinking package which we used to implement these tests does not support the fitting of zero-inflated negative binomial models for comparison to those we report for experiment 3 in Uganda. Negative binomial models were in agreement with the over-dispersed Poisson models presented in main text.

Table A: A summary of posterior distributions for the coefficients estimated by analysis of experiment 1 data using a negative binomial model. Values in brackets are those for the equivalent over-dispersed Poisson model presented in Table 1 of main text.

|  | Mean | SD | 5.50% HPDI | 94.50% HPDI | N_effective_ | R̂ |
| --- | --- | --- | --- | --- | --- | --- |
| α | 3.42 (3.39) | 0.34 (0.36) | 2.91 (2.85) | 3.94 (3.92) | 6754 (8721) | 1 (1) |
| β_Typ.Blue_ | -0.42 (-0.42) | 0.13 (0.12) | -0.62 (-0.62) | -0.21 (-0.22) | 17934 (15125) | 1 (1) |
| β_Vest.SP23(P)_ | -0.07 (-0.08) | 0.12 (0.12) | -0.27 (-0.27) | 0.12 (0.12) | 17649 (13876) | 1 (1) |
| β_Violet_ | -0.04 (-0.04) | 0.12 (0.12) | -0.23 (-0.23) | 0.16 (0.15) | 18373 (14710) | 1 (1) |
| σ_day_ | 0.32 (0.32) | 0.09 (0.09) | 0.20 (0.20) | 0.47 (0.47) | 7616 (9049) | 1 (1) |
| σ_site_ | 0.58 (0.58) | 0.30 (0.31) | 0.26 (0.26) | 1.15 (1.15) | 8904 (10821) | 1 (1) |
| σ_rep_ | N/A (0.28) | N/A (0.05) | N/A (0.21) | N/A (0.36) | N/A (8759) | N/A (1) |
| scale | 13.26 (N/A) | 3.73 (N/A) | 8.10 (N/A) | 19.80 (N/A) | 15663 (N/A) | 1 (N/A) |

*SD = standard deviation; HPDI = highest posterior density interval; N_effective_ (= the effective number of samples) and R̂ are convergence and efficiency diagnostics for Markov chains used in model fitting.*

Table B: Future target performance predicted by analysis of experiment 1 data using a negative binomial model. Values in brackets are those for the equivalent over-dispersed Poisson model presented in Table 2 of main text.

|  | Percentage of deployments in which expected catch exceeds … | | |  |
| --- | --- | --- | --- | --- |
| Target | (i) Typ. Blue | (ii) Vest. ZF | (iii) Vest. SP23 (P) | (iv) Violet |
| Typ. Blue |  | 0% (0%) | 0% (0%) | 0% (0%) |
| Vest. ZF | 100% (100%) |  | 73% (73%) | 63% (64%) |
| Vest. SP23 (P) | 100% (100%) | 27% (27%) |  | 39% (39%) |
| Violet | 100% (100%) | 37% (36%) | 61% (61%) |  |

*Table shows the percentage of 10,000 simulated catches in which the expected catch (μ for a negative binomial model, or λ for a Poisson model) of the target in the row heading exceeded that in the column heading for the average catch replicate (i.e. excluding variability in catches between replicates).*

Table C: A summary of posterior distributions for the coefficients estimated by analysis of experiment 2 data using a negative binomial model. Values in brackets are those for the equivalent over-dispersed Poisson model presented in Table 3 of main text.

|  | Mean | SD | 5.50% HPDI | 94.50% HPDI | N_effective_ | R̂ |
| --- | --- | --- | --- | --- | --- | --- |
| α | 2.95 (2.89) | 0.19 (0.19) | 2.64 (2.58) | 3.25 (3.19) | 9439 (11470) | 1 (1) |
| β_Violet4.5%_ | 0.07 (0.10) | 0.14 (0.14) | -0.14 (-0.12) | 0.29 (0.32) | 18525 (16255) | 1 (1) |
| β_Violet7%_ | 0.24 (0.25) | 0.13 (0.14) | 0.03 (0.04) | 0.46 (0.47) | 18893 (15957) | 1 (1) |
| β_Violet9%_ | 0.19 (0.21) | 0.13 (0.14) | -0.02 (-0.01) | 0.40 (0.43) | 19072 (15828) | 1 (1) |
| σ_day_ | 0.33 (0.32) | 0.09 (0.09) | 0.21 (0.19) | 0.49 (0.48) | 8891 (8900) | 1 (1) |
| σ_site_ | 0.38 (0.38) | 0.15 (0.14) | 0.21 (0.21) | 0.64 (0.64) | 8720 (11920) | 1 (1) |
| σ_rep_ | N/A (0.31) | N/A (0.06) | N/A (0.23) | N/A (0.41) | N/A (8078) | N/A (1) |
| scale | 11.57 (N/A) | 3.49 (N/A) | 6.81 (N/A) | 17.72 (N/A) | 15564 (N/A) | 1 (N/A) |

Table D. Future target performance predicted by analysis of experiment 2 data using a negative binomial model. Values in brackets are those for the equivalent over-dispersed Poisson model presented in Table 4 of main text.

|  | Percentage of deployments in which expected catch exceeds… | | |  |
| --- | --- | --- | --- | --- |
| Target | (i) Vest. ZF | (ii) Violet 4.5% (P) | (iii) Violet 7% (P) | (iv) Violet 9% (P) |
| Vest. ZF |  | 29% (23%) | 3% (3%) | 8% (7%) |
| Violet 4.5% (P) | 71% (77%) |  | 10% (13%) | 20% (21%) |
| Violet 7% (P) | 97% (97%) | 90% (87%) |  | 67% (62%) |
| Violet 9% (P) | 92% (93%) | 80% (79%) | 33% (38%) |  |

Table E: A summary of posterior distributions for the coefficients estimated by analysis of experiment 3 data using a negative binomial model. Values in brackets are those for the equivalent non-zero-inflated over-dispersed Poisson model presented in Table 5 of main text.

|  | Mean | SD | 5.50% HPDI | 94.50% HPDI | N_effective_ | R̂ |
| --- | --- | --- | --- | --- | --- | --- |
| α | 0.34 (0.15) | 0.61 (0.63) | -0.61 (-0.84) | 1.27 (1.09) | 9614 (11715) | 1 (1) |
| β_Violet_ | 0.09 (0.07) | 0.35 (0.40) | -0.47 (-0.56) | 0.65 (0.69) | 22437 (17541) | 1 (1) |
| β_Typ.Blue_ | -0.39 (-0.45) | 0.38 (0.43) | -0.99 (-1.15) | 0.21 (0.21) | 23082 (17626) | 1 (1) |
| σ_day_ | 0.52 (0.49) | 0.29 (0.29) | 0.08 (0.06) | 1.00 (0.99) | 5807 (5642) | 1 (1) |
| σ_site_ | 0.83 (0.82) | 0.42 (0.44) | 0.31 (0.28) | 1.61 (1.63) | 14517 (14814) | 1 (1) |
| σ_rep_ | N/A (0.69) | N/A (0.28) | N/A (0.21) | N/A (1.13) | N/A (4981) | N/A (1) |
| scale | 5.24 (N/A) | 4.57 (N/A) | 1.10 (N/A) | 14.39 (N/A) | 11167 (N/A) | 1 (N/A) |

Table F: Future target performance predicted by analysis of experiment 3 data using a negative binomial model. Values in brackets are those for the equivalent non-zero-inflated over-dispersed Poisson model presented in Table 6 of main text.

|  | Percentage of deployments in which expected catch exceeds … | | |
| --- | --- | --- | --- |
| Target | (i) Typ. Blue | (ii) Vest. ZF | (iii) Violet |
| Typ. Blue |  | 14% (14%) | 10% (10%) |
| Vest. ZF | 86% (86%) |  | 40% (43%) |
| Violet | 90% (90%) | 60% (57%) |  |

Table G: A summary of posterior distributions for the coefficients estimated by analysis of new data collected in Kenya incorporating priors informed by analysis of previously published data [2], using a negative binomial model. Values in brackets are those for the equivalent over-dispersed Poisson model presented in Table 8 of main text.

|  | Mean | SD | 5.50% HPDI | 94.50% HPDI | N_effective_ | R̂ |
| --- | --- | --- | --- | --- | --- | --- |
| α | 3.00 (2.96) | 0.34 (0.34) | 2.45 (2.41) | 3.55 (3.51) | 11314 (14334) | 1 (1) |
| β_R7p_ | -1.98 (-1.97) | 0.37 (0.37) | -2.57 (-2.57) | -1.39 (-1.38) | 25915 (22614) | 1 (1) |
| β_R7y_ | 2.22 (2.22) | 0.47 (0.47) | 1.48 (1.47) | 2.96 (2.97) | 15305 (21834) | 1 (1) |
| β_R8y_ | -0.87 (-0.89) | 0.25 (0.25) | -1.27 (-1.30) | -0.47 (-0.49) | 18057 (18578) | 1 (1) |
| σ_day_ | 0.32 (0.32) | 0.06 (0.06) | 0.24 (0.23) | 0.42 (0.41) | 8359 (8699) | 1 (1) |
| σ_site_ | 0.40 (0.40) | 0.11 (0.11) | 0.26 (0.26) | 0.60 (0.60) | 6797 (9421) | 1 (1) |
| σ_rep_ | N/A (0.28) | N/A (0.03) | N/A (0.23) | N/A (0.34) | N/A (10319) | N/A (1) |
| scale | 12.98 (N/A) | 2.80 (N/A) | 9.03 (N/A) | 17.88 (N/A) | 17914 (N/A) | 1 (N/A) |

Table H. Future target performance predicted by analysis of new data collected in Kenya incorporating priors informed by analysis of previously published data [2], using a negative binomial model. Values in brackets are those for the equivalent over-dispersed Poisson model presented in Table 9 of main text.

|  | Percentage of deployments in which expected catch exceeds … | | |
| --- | --- | --- | --- |
| Target | (i) Typ. Blue | (ii) ‘Ave. Vest.’ | (iii) ‘Ave. violet’ |
| Typ. Blue |  | 0% (0%) | 0% (0%) |
| ‘Average Vest.’ | 100% (100%) |  | 4% (4%) |
| ‘Average violet’ | 100% (100%) | 96% (96%) |  |

Table I: A summary of posterior distributions for the coefficients estimated by analysis of data collected in Uganda incorporating priors from the analysis of data collected in Kenya that itself used informative priors, using a negative binomial model. Values in brackets are those for the equivalent non-zero-inflated over-dispersed Poisson model presented in Table 10 of main text.

|  | Mean | SD | 5.50% HPDI | 94.50% HPDI | N_effective_ | R̂ |
| --- | --- | --- | --- | --- | --- | --- |
| α | 0.21 (0.03) | 0.72 (0.71) | -0.93 (-1.11) | 1.33 (1.14) | 15358 (12101) | 1 (1) |
| β_R7p_ | -2.00 (-2.01) | 0.39 (0.39) | -2.62 (-2.62) | -1.39 (-1.39) | 40184 (37770) | 1 (1) |
| β_R7y_ | 2.21 (2.20) | 0.49 (0.49) | 1.42 (1.42) | 2.99 (2.98) | 34078 (32072) | 1 (1) |
| β_R8y_ | -0.90 (-0.90) | 0.28 (0.29) | -1.34 (-1.36) | -0.45 (-0.44) | 46696 (42201) | 1 (1) |
| σ_day_ | 0.53 (0.51) | 0.29 (0.30) | 0.09 (0.07) | 1.01 (1.00) | 6282 (4602) | 1 (1) |
| σ_site_ | 0.83 (0.82) | 0.42 (0.43) | 0.33 (0.29) | 1.62 (1.62) | 15853 (15675) | 1 (1) |
| σ_rep_ | N/A (0.60) | N/A (0.27) | N/A (0.15) | N/A (1.03) | N/A (3965) | (N/A) 1 |
| scale | 6.05 (N/A) | 4.90 (N/A) | 1.28 (N/A) | 15.80 (N/A) | 14974 (N/A) | 1 (N/A) |

Table J. Future target performance estimated by analysis of new data collected in Uganda incorporating priors from the analysis of data collected in Kenya that itself used informative priors, using a negative binomial model. Values in brackets are those for the equivalent non-zero-inflated over-dispersed Poisson model presented in Table 11 of main text.

|  | Percentage of deployments in which expected catch exceeds … | | |
| --- | --- | --- | --- |
| Target | (i) Typ. Blue | (ii) ‘Ave. Vest.’ | (iii) ‘Ave. violet’ |
| Typ. Blue |  | 0% (0%) | 0% (0%) |
| ‘Average Vest.’ | 100% (100%) |  | 12% (12%) |
| ‘Average violet’ | 100% (100%) | 88% (88%) |  |

**S1.2 Posterior predictive checks for Bayesian models**

On the following pages we present posterior predictive checks for validation of the models presented in main text. Figs A and B illustrate the distribution or predictions, and the data actually recorded, for each case in our dataset.


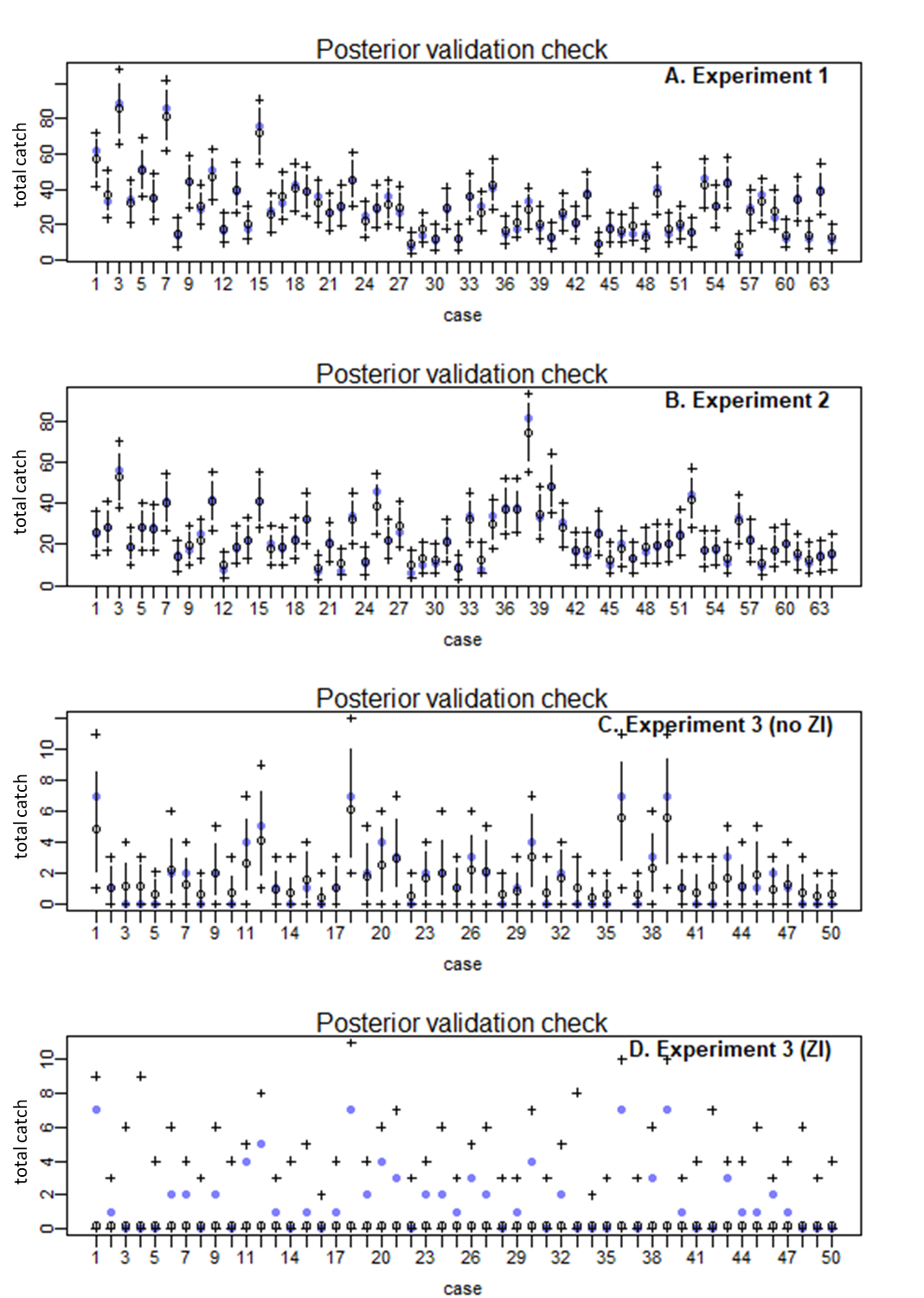


Fig. A: Posterior predictive checks for Bayesian analyses of each of our experiments individually, using regularising priors. Blue dots indicate each catch observation. Open circles are the posterior mean predictions, and lines are the 89% posterior intervals of the mean predictions. Plus symbols indicate the 89% interval of predicted catches. In all cases, recorded catches lie within the 89% interval of predictions. ZI = zero-inflation.


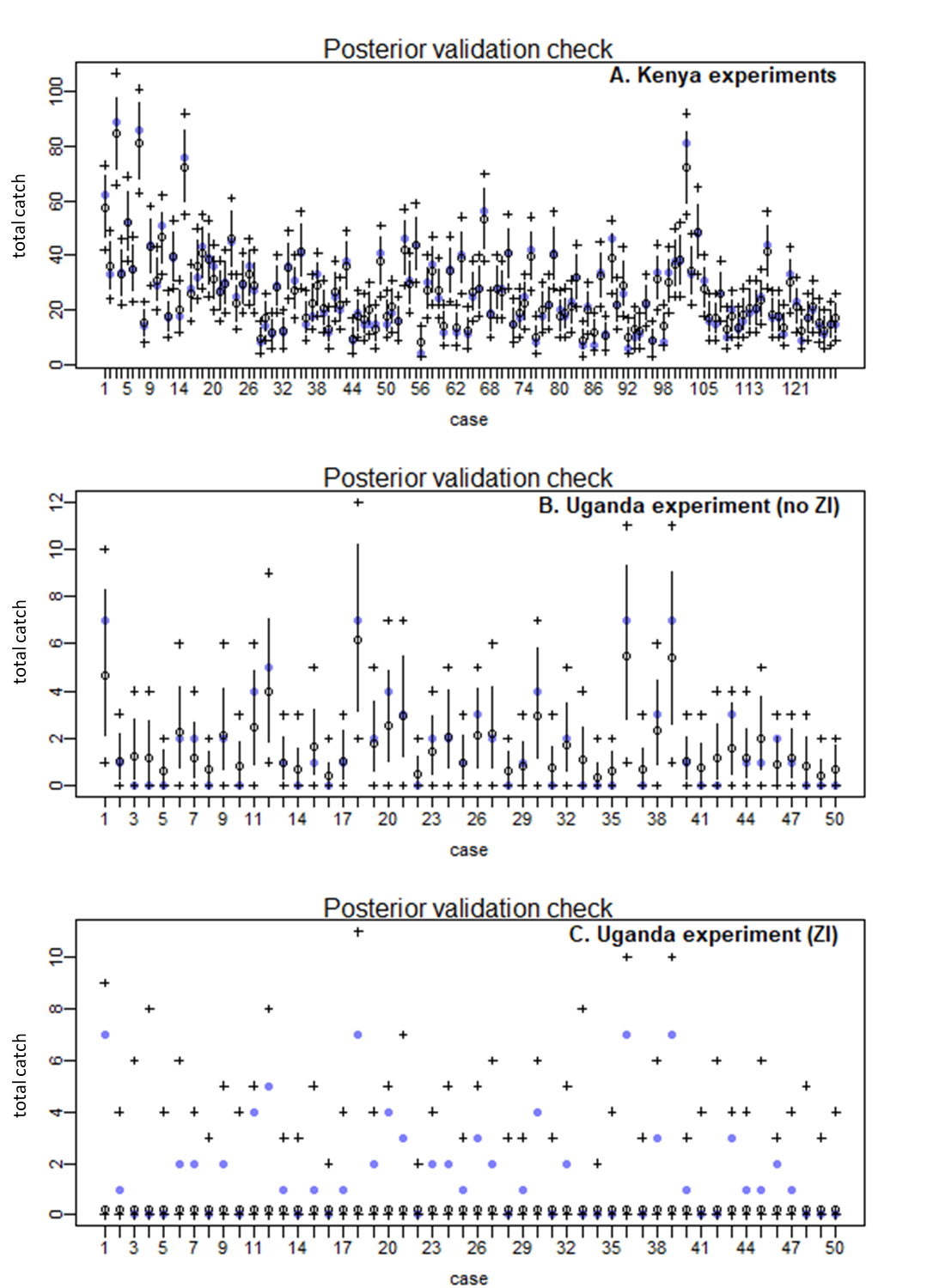


Fig. B: Posterior predictive checks for Bayesian analyses of our data using photoreceptor responses as predictors, and priors informed by previous work. Blue dots indicate each catch observation. Open circles are the posterior mean predictions, and lines are the 89% posterior intervals of the mean predictions. Plus symbols indicate the 89% interval of predicted catches. In all cases, recorded catches lie within the 89% interval of predictions. ZI = zero-inflation.

**S1.3 Equivalent analyses using generalised linear mixed models (GLMMs)**

In this section we report additional analyses of the three experiments in our manuscript using Generalised Linear Mixed Models implemented in the glmmTMB package for R [3]. We report models assuming a negative binomial distribution of catches, but note that inferences from over-dispersed Poisson models were identical (not shown). Posthoc LSD tests were conducted using the emmeans package for R [4].

**S1.3.1 Experiment 1: Vest. ZF vs Vest. SP23(P) vs Violet vs Typ. Blue, in Kenya**

In experiment 1, there was a significant effect of target type on daily tsetse catch (Negative binomial/log link GLMM: Wald Χ^2^_3_=15.567, p=0.001). The catch of Typ. Blue was significantly less than that of any other target (posthoc LSD tests, p<0.01); there were no significant differences between the catches of Violet, Vest. ZF, and Vest. SP23(P) targets (posthoc LSD tests, p>0.50).

**S1.3.2 Experiment 2: Vest. ZF vs Violet 4.5%(P) vs Violet 7%(P) vs Violet 9%(P), in Kenya**

In experiment 2, there was no significant effect of target type on daily tsetse catch (Negative binomial/log link GLMM: Wald Χ^2^_3_=4.575, p=0.206).

**S1.3.3 Experiment 3: Vest. ZF vs Violet vs Typ. Blue, in Uganda**

In experiment 3, there was no significant effect of target type on daily tsetse catch using negative binomial models with or without zero-inflation (Negative binomial/log link GLMM: Wald Χ^2^_2_=1.103, p=0.576; Zero-inflated negative binomial/log link GLMM: Wald Χ^2^_2_=0.874, p=0.646).

**References**

1. McElreath R. Statistical rethinking: A Bayesian course with examples in R and Stan. Boca Raton, FL: CRC Press; 2016.

2. Lindh JM, Goswami P, Blackburn R, Arnold SEJ, Vale GA, Lehane MJ, et al. Optimizing the colour and fabric of targets for the control of the tsetse fly *Glossina fuscipes fuscipes*. PLoS Negl Trop Dis. 2012;6(5):e1661.

3. Brooks ME, Kristensen K, van Benthem KJ, Magnussen A, Berg CW, Nielsen A, et al. glmmTMB balances speed and flexibility among packages for zero-inflated generalized linear mixed modeling. The R Journal. 2017;9(2):378-400.

4. Lenth R. emmeans: estimated marginal means, aka least-squares means. R package version 1.3.2. <https://CRAN.R-project.org/package=emmeans>; 2019.
